# The six metal binding domains in human copper transporter, ATP7B: molecular biophysics and disease-causing mutations

**DOI:** 10.1007/s10534-017-0058-2

**Published:** 2017-10-23

**Authors:** Candan Ariöz, Yaozong Li, Pernilla Wittung-Stafshede

**Affiliations:** 1 Department of Biology and Biological Engineering, Division of Chemical Biology, Chalmers University of Technology, Kemigården 4, 412 96 Gothenburg, Sweden; 2 Department of Chemistry, Umeå University, Kemihuset A, Linnaeus väg 10, 901 87 Umeå, Sweden

**Keywords:** Wilson disease, ATP7B, Atox1, Metal-binding domains, Cu transport, Disease-causing mutations

## Abstract

Wilson Disease (WD) is a hereditary genetic disorder, which coincides with a dysfunctional copper (Cu) metabolism caused by mutations in ATP7B, a membrane-bound P_1B_-type ATPase responsible for Cu export from hepatic cells. The N-terminal part (~ 600 residues) of the multi-domain 1400-residue ATP7B constitutes six metal binding domains (MBDs), each of which can bind a copper ion, interact with other ATP7B domains as well as with different proteins. Although the ATP7B’s MBDs have been investigated in vitro and in vivo intensively, it remains unclear how these domains modulate overall structure, dynamics, stability and function of ATP7B. The presence of six MBDs is unique to mammalian ATP7B homologs, and many WD causing missense mutations are found in these domains. Here, we have summarized previously reported in vitro biophysical data on the MBDs of ATP7B and WD point mutations located in these domains. Besides the demonstration of where the research field stands today, this review showcasts the need for further biophysical investigation about the roles of MBDs in ATP7B function. Molecular mechanisms of ATP7B are important not only in the development of new WD treatment but also for other aspects of human physiology where Cu transport plays a role.

**Electronic supplementary material:**

The online version of this article (doi:10.1007/s10534-017-0058-2) contains supplementary material, which is available to authorized users.

## Introduction

Copper (Cu) is an essential metal ion that plays key roles in metabolic processes acting as a reactive cofactor in proteins (e.g., connective tissue formation, tryptophan synthesis, iron bioavailability, respiration, free radical scavenging, pigmentation, and neurotransmitter synthesis) (Letelier et al. 2005; Matson Dzebo et al. 2016; Polishchuk et al. 2014; Prashanth et al. 2015). Because of its ability to switch between Cu (I) and Cu (II), this metal is often used by a number of copper-dependent enzymes in electron-transfer reactions (Festa and Thiele 2011; Pham et al. 2013; Yu et al. 2017a). The high redox potential of copper ions allow them, when in free form, to generate hydroxyl radicals (.OH) that can damage the structures of proteins, nucleic acids and lipids of cells (Festa and Thiele 2011). Therefore, free copper ions and/or their abnormal levels can be toxic for cells so that their distribution is tightly regulated via dedicated protein systems that facilitate its uptake, efflux and delivery to Cu-dependent enzymes (DiDonato et al. 2000; Kumar et al. 2017; Matson Dzebo et al. 2016; Mondol et al. 2016).

In humans, Cu enters the cell via the membrane-bound high-affinity copper uptake protein 1 (Ctr1) (Gupta and Lutsenko 2009; Wang et al. 2011). How Cu(II) in the blood is reduced to Cu(I) remains uncertain but the possible involvement of a membrane-bound copper reductase close to the Ctr1 was mentioned (Knöpfel and Solioz 2002; Lutsenko et al. 2007; Shatwell et al. 1996; Taylor et al. 2005). Nevertheless, after entering the cytoplasm copper is presumably in the reduced Cu(I) form because the cytoplasm is a highly-reducing environment due to its high glutathione (GSH) content (Kaplan and Maryon 2016; Lopez-Mirabal and Winther 2008). Therefore herein, the abbreviation “Cu” refers “Cu(I)” unless otherwise stated. In the cytoplasm, there are at least three distinct pathways of copper transport for metallation of Cu-dependent proteins/enzymes. One pathway involves the copper chaperone for superoxide dismutase (CCS) that carries Cu ions to Cu/Zn superoxide dismutase 1 (SOD1), the primary cytoplasmic scavenger of superoxide radical (.O_2_^−^) (Matson Dzebo et al. 2016; Rakhit and Chakrabartty 2006). Another route is to the mitochondria: here, although there is much unknown, somehow the cytochrome c oxidase copper chaperone, Cox17, transfers Cu via the help of additional proteins (e.g., Sco1, Sco2, Cox11) to cytochrome c oxidase (COX), the final electron acceptor in the mitochondrial electron transport chain (Horn and Barrientos 2008). In the third and general cytoplasmic pathway for Cu, Atox1 (alias: HAH1), a 68-residue cytoplasmic Cu chaperone, transfers Cu to ATP7A (Menke’s disease protein) or ATP7B (Wilson disease protein), two homologous membrane proteins in the trans-Golgi network (TGN) (Banci et al. 2008; Barnes et al. 2009; Hamza et al. 1999). Following this, Cu ions are either used for the biosynthesis of various copper-dependent enzymes such as tyrosinase, lysyl oxidase and ceruloplasmin in the secretory pathway, or (if there is excess Cu) sequestered in cytoplasmic membrane vesicles for excretion from the cell to the bile (Cater et al. 2006; Dmitriev et al. 2006).

ATP7A and ATP7B are P_1B_-type ATPases, which use the energy released from ATP hydrolysis to transport Cu across Golgi membranes (Jayakanthan et al. 2017; Lutsenko et al. 2008; Yatsunyk and Rosenzweig 2007; Yu et al. 2017b). Although they share a close homology (67% amino acid similarity) (Yatsunyk and Rosenzweig 2007), they can be distinguished by unique properties (Hamza et al. 1999; Mercer et al. 2003). For example, ATP7A is primarily expressed in non-hepatic tissues such as intestines, brain, heart etc. whereas ATP7B is mostly found in liver and kidney, but also detected with lower levels in the lung, placenta and brain (Bunce et al. 2006; Cater et al. 2004; DiDonato et al. 2000). ATP7A is mostly responsible for Cu absorption from intestinal enterocytes to the blood and function-impairing mutations of this protein cause Menke’s disease (MD). As a result, intestinal cells accumulate excess amount of Cu, and thereby the blood-Cu level is decreased, leading to decreased Cu delivery to other tissues (Keller et al. 2012; Lutsenko et al. 2007). Patients with MD suffer from connective tissue abnormalities, lack of pigmentation, diminished functions of Cu-dependent enzymes and tortuosity of blood vessels due to the impaired delivery of Cu especially to the brain and the majority of these patients die at early childhood (Banci et al. 2009b). On the other hand, ATP7B is mostly responsible for Cu efflux from hepatocytes (Dmitriev et al. 2006; Keller et al. 2012) and impairment of ATP7B function due to mutations results in an autosomal recessive disorder known as Wilson disease (WD). The main biochemical phenotype for WD is a chronic copper toxicosis where hepatocytes fail to remove excess copper and is observed by clinical symptoms such as hepatic abnormalities, neurological defects, more commonly psychiatric/behavioral symptoms (Hedera 2017), and eventually liver failure which can cause death when untreated (Chesi et al. 2016; DiDonato et al. 2002; Yu et al. 2017c). For an adult, basic daily copper requirement is ~1–2 mg and under normal circumstances Cu overload is dissipated by ATP7B and maintained at levels less than 50 µg Cu/g dry liver. However, in WD patients hepatic Cu levels can reach up to 250–3000 µg Cu/g dry liver (Das and Ray 2006; Suzuki et al. 2002). To reduce it to normal levels, there are different strategies currently used as WD treatment involving removal of excess Cu by the administration of Cu chelators such as D-penicillamine (D-PCA; with very severe side effects such as immunological disturbances, skin defects, joint disorders and worsened neurological manifestations), Trientine (with lower side effects compared to D-penicillamine) and the novel chelating agents such as tetrathimolybdate (TTM; blocks Cu absorption) or choline tetrathiomolybdate (CTTM; a more stable salt formulation of tetrathiomolybdate) that are being currently evaluated (Chang et al. 2013; Schilsky 2014; Weiss 2016). After Cu detoxification, re-accumulation of Cu in tissues are prevented by a “maintenance therapy” throughout life in which patients use lowered dosages (25–33% of the initial dose) of above mentioned chelators/agents or zinc salts (prevents intestinal Cu absorption but increases metallothionein, an heavy-metal scavenger) but also maintain a low copper diet (Schilsky 2014). However, some of the agents described above have been reported to cause significant problems when they are used long-term (Roberts et al. 2008), therefore new drugs with less side effects are desired. Understanding how disease-causing mutations change ATP7B function at the molecular level is of great importance for developing new medication strategies for this disease.

**Fig. 1:**
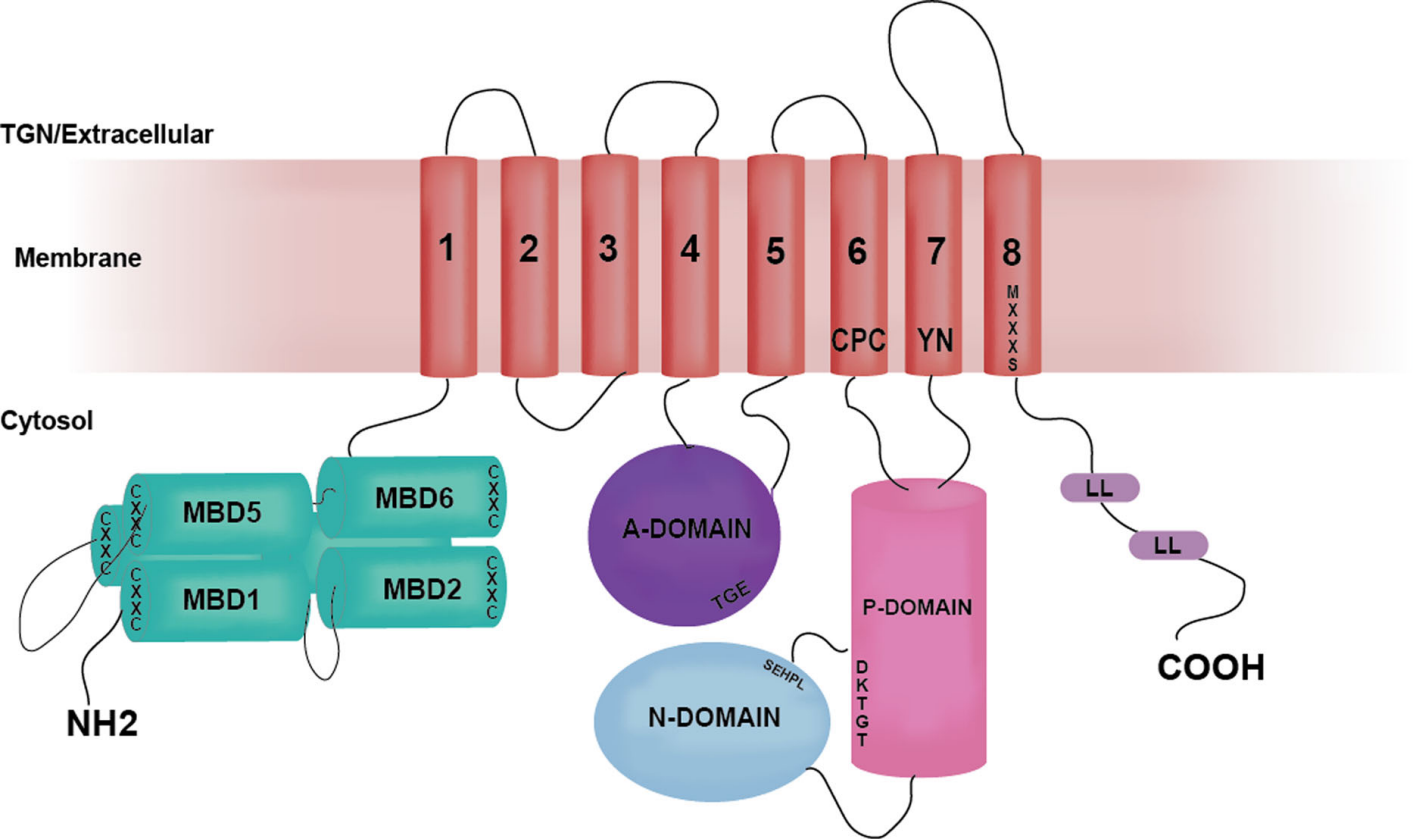
The structural organization of ATP7B. Cartoon illustrates the major functional domains and important motifs for Cu-ATPases. ATP7B and other P-type ATPases consist of three cytoplasmic domains; nucleotide binding domain (N-domain, blue), phosphorylation domain (P-domain, pink), actuator/dephosphorylation domain (A-domain, purple). A combination of N- and P-domains are usually referred as ATP-binding domain. ATP7B hydrolyzes ATP and the invariant aspartate (D) located at the **D**KTGT motif accepts γ-phosphate from ATP during the catalytic cycle forming a transient phosphorylated intermediate (E1P). SE**H**PL is a conserved motif among all ATPases transporting transition metals (P_1_-type ATPases or CPx-ATPases) and includes H1069 which is believed be involved in correct positioning of ATP prior to transfer of the γ-phosphate (Tsivkovskii et al. 2003). TGE motif in the A-domain is responsible for the removal of γ-phosphate from the DKTGT motif (dephosphorylation). The N-terminal contains six metal-binding domains (MBD1-MBD6, cyan) with conserved CxxC Cu-binding motifs. The transmembrane domain (TMD) encompasses eight transmembrane helices and position of residues that are predicted to be involved in Cu-coordination within the membrane are depicted as CPC, YN and MXXXS. The two leucine motifs (1459LL and 1487LL) located at the C-terminal have been shown to be essential for endocytosis and/or TGN relocalization from plasma membrane

ATP7B has a basic P-type ATPase architecture (Banci et al. 2009b) with a cytosolic region composed of phosphorylation (P-), ATP-binding (N-) and actuator/dephosphorylation (A-) domains and a membrane part where eight transmembrane helices (transmembrane domains, TMDs) form an intramembranous Cu channel. A unique structural feature of this multi-domain protein, is the presence of a large, cytosolic N-terminal tail containing six 70-aa long independently folded Cu (I) binding domains (hereinafter metal binding domains, MBDs) (Fig. 1) (Banci et al. 2009b; LeShane et al. 2010; Mondol et al. 2016). MBDs are named from the N-terminal, with MBD1 being the first domain from the N-terminus and MBD6 the domain closest to the membrane-spanning part of ATP7B. The domains are connected by peptide linkers of various length (Fig. 3C). The complete N-terminus is about 630 residues long (Banci et al. 2009a) where roughly 200 residues belong to the inter-domain linkers. The longest linker is located between MBD4 and MBD5, consisting of 76 residues. The length of other linkers is relatively short: namely, 29 residues between MBD3-MBD4, 24 residues between MBD2-MBD3, 13 residues between MBD1-MBD2 and 6 residues between MBD5-MBD6 (Fig. 3C). The basic P-type ATPase architecture does not change from bacteria to humans but the number of MBDs greatly differentiate between phylogenetic families. Mammals, birds and reptiles have six MBDs; nematodes and insects contain four MBDs, whereas bacteria or lower eukaryotes (e.g. yeast) contain only one or two MBDs (Yu et al. 2017a). The increasing number of MBDs from bacteria to humans can be seen as an evolutionary advantage for human ATPases to fine-tune their activities. Such regulatory function can be fulfilled via interactions between MBDs and other domains of ATP7B (or other proteins) (Mondol et al. 2016; Sharma and Rosato 2009). Although some in vitro work have suggested the presence of domain–domain communication/s between MBDs and MBDs with the core of the ATP7B, the exact roles of these communications in vivo remain unknown. It is even not clear if MBDs are on the Cu-route towards the membrane channel or merely dead-end Cu reservoirs. Nonetheless, disease-causing mutations can be found in the ATP7B MBDs and thus these domains must critically impact normal Cu-transport activity. In contrast, MD mutations have not been reported to be positioned in any MBDs of ATP7A (Hamza et al. 1999), except for E628V and K633R, both of which are located in MBD6 of ATP7A (Skjorringe et al. 2017).

ATP7B adheres to the working mechanism of all P-type ATPases, the Post-Albers catalytic cycle where a phosphorylation-dephosphorylation cascade occur with four principal conformations described as E1 (high affinity), E1P, E2 (low affinity) and E2P states (Gourdon et al. 2012; Skjorringe et al. 2017). In the catalytic cycle of ATP7B, ATP binding to N-domain and Cu binding to the CPC motif (in TMD6) (E1 state), are followed by the transient phosphorylation of the D1027 residue located at the P-domain upon the transfer of the γ-phosphate from ATP. This transfer (E1P state) enables Cu to be occluded from the intramembrane region to the Golgi lumen and is followed by a conformational change within ATP7B structure which allows the Cu to be translocated across the membrane (E2P state). This is followed by dephosphorylation of the invariant D1027 residue by the A-domain (E2 state) (Banci et al. 2009b). These steps are altogether coupled to conformational changes within ATP7B and it is proposed that alterations of domain–domain interactions, notably involving the MBDs, are of high relevance for ATPase activity (Banci et al. 2009a; Huang et al. 2014; Tsivkovskii et al. 2001). ATP7B normally resides in trans-Golgi network (TGN) of hepatocytes under low Cu conditions (Polishchuk et al. 2014) but as Cu levels increase, it exists TGN and re-localizes into cytoplasmic membrane vesicles to sequester excess cytosolic Cu. Later, these vesicles move close to the plasma membrane where excess Cu is extruded from the cell via vesicle-mediated exocytosis (Wu et al. 2015). As Cu levels are depleted, ATP7B returns from vesicles to the TGN to continue its role in Cu-loading of proteins (Huang et al. 2014).

To display what is known today about ATP7B MBDs from a biophysical viewpoint, here we have summarized previously reported results for various constructs of the six MBDs of ATP7B (Supplementary Table S1) and also gathered the limited biophysical information of known WD point mutations that are located in the six human MBDs (Table 1).

**Table 1:** Wilson disease-causing mutations localized in MBDs of ATP7B and their reported effects on ATP7B function

MBD Domain	Mutation	Impact of mutation	References
MBD1	N41S	Partially defective trafficking	Braiterman et al. (2009)
	M67V	Reported as disease-causing mutation. No available information	Mukherjee et al. (2014)
	G85V	Decreased interaction with Atox1 Normal ATP7B trafficking	Hamza et al. (1999)
		Significant residual copper export capacity in HEK293T cells Reduced protein expression and mislocalization to the ER Normal interaction with Atox1/increased interaction with COMMD1 Co-localization both into ER and TGN	van den Berghe et al. (2009)
		Increased interaction with COMMD1	de Bie et al. (2007)
		Completely defective catalytic and transport activity/reduced phosphorylation	Huster et al. (2012)
MBD2	R136G	Reported as disease-causing mutation. No available information	Mukherjee et al. (2014)
	R198G	Reported as disease-causing mutation. No available information	Dong et al. (2016)
MBD3	G333R	Located at the linker region between MBD3 and MBD4/no change in protein stability	Hinz (2014)
MBD4	I390V	Reported as disease-causing mutation. No other available information	Dong et al. (2016)
MBD5	S406A	Normal Cu transport activity/hyperphosphorylation	Huster et al. (2012)
	V456L	Partial Cu transport activity/hyperphosphorylation	
	A476T	Reported as disease-causing mutation. No available information	Lin et al. (2010)
	A486S	Slightly increased interaction with COMMD1	de Bie et al. (2007)
	L492S	Decreased interaction with Atox1 Does not interfere with ATP7B trafficking	Hamza et al. (1999)
		Completely defective catalytic and transport activity/reduced phosphorylation	Huster et al. (2012)
		Increased interaction with COMMD1	de Bie et al. (2007)
	G515V	Reported as disease-causing mutation. No available information	Dong et al. (2016)
	V519M	Reported as disease-causing mutation. No available information	Kroll et al. (2006)
	Y532H	Normal Cu transport activity/normal protein expression	Hsi et al. (2008)
		Slightly increased interaction with COMMD1	de Bie et al. (2007)
	E541K	Reported as disease-causing mutation. No available information	Bugbee et al. (2010)
	L549P	Reported as disease-causing mutation. No available information	Abdelghaffar et al. (2008)
			Nagasaka et al. (2012)
MBD6	T587M	Reported as disease-causing mutation. No available information	Dong et al. (2016)
	G591S	Reported as disease-causing mutation. No available information	Mukherjee et al. (2014)
	G591D	Decreased interaction with Atox1. Does not interfere with ATP7B trafficking	Pilankatta et al. (2011)
		Normal phosphorylation but its Cu-response was impaired so MBD1-6 can be important for Cu-induced response	Vanderwerf et al. (2001)
		Increased interaction with COMMD1	de Bie et al. (2007)
		Does not interfere with ATP7B trafficking	Hamza et al. (1999)
MBD6	A595T	Reported as disease-causing mutation. No available information	Mukherjee et al. (2014)
	A604P	Increased interaction with COMMD1	de Bie et al. (2007)
	R616Q	Reported as disease-causing mutation. No available information	Loudianos et al. (2003)
			Todorov et al. (2005)
			Mak et al. (2008)
		Slightly impaired Cu transport activity/normal Cu-dependent trafficking of ATP7B/normal protein expression/might cause structural instability	Scvortova (2013)
	R616W	Completely defective catalytic and transport activity/hyperphosphorylation	Huster et al. (2012)
	G626A	Partial catalytic and transport activity/reduced phosphorylation	
		Normal protein expression/normal Cu-transport activity/normal Cu responsive trafficking	Braiterman et al. (2014)
		Normal Cu transport activity/is situated in a helix adjacent to the CxxC motif and may have some impact upon copper binding	Hsi et al. (2008)

**Table 2:** PDB entries for reported high-resolution structures of the MBDs in ATP7B. All PDB structures represent the metal-free state of the related protein. Structures were determined by solution NMR and protein domains (single or double domain constructs) were recombinantly expressed in *E. coli*

PDB no	Domain	Organism	Ref
2N7Y	MBD1	Homo sapiens	Yu et al. (2016)
2LQB	MBD2	Homo sapiens	Dolgova et al. (2013)
2ROP	MBD3–4	Homo sapiens	Banci et al. (2008)
2EW9	MBD5–6	Homo sapiens	Achila et al. (2006)

### Cu coordination in ATP7B MBDs

Several in vitro and in silico studies have investigated Cu coordination of MBDs and its effects on the protein structure using individual MBD domains or multi-domain MBD constructs (Supplementary Table S1). Based on previous work, the overall structure of each domain is similar to each other and to the MBDs of ATP7A and to that of Atox1 (Achila et al. 2006; Banci et al. 2008; Fatemi et al. 2010; Walker et al. 2004). All MBDs have a ferredoxin-like fold (Achila et al. 2006; Banci et al. 2008) where the Cu-binding site is situated in a solvent-exposed loop between β1-strand and α1-helix (Braiterman et al. 2015; LeShane et al. 2010). Here, each MBD includes a strictly conserved Cu-binding motif GMX_1_CX_2_X_3_CV, known also as the Cu site, which can bind a single Cu(I) ion via di-cysteine coordination of the two sulfur residues in cysteines (C_1_ and C_2_) (DiDonato et al. 1997, 2000, 2002; Hussain et al. 2009; Larin et al. 1999; Lutsenko et al. 1997; Niemiec et al. 2014; Rodriguez-Granillo et al. 2009; Walker et al. 2004). This coordination geometry does not change in any of the MBDs and was shown with X-ray absorption spectroscopy (XAS) to be very similar to that for Cu in Atox1 (Ralle et al. 1998). Both in vitro and in silico work with different constructs of MBDs (e.g. MBD1-6 as single domains or as entire MBD1-6) (DiDonato et al. 2002; Rodriguez-Granillo et al. 2009; Walker et al. 2004) showed that increasing Cu concentration does not affect Cu coordination. However, an XAS/EXAFS analysis of MBD1-6 construct revealed that Cu sites in each MBD can be distorted from the ideal linear geometry of a two-coordinate Cu(I) center to a geometry with a S–Cu–S angle between 120° and 180° maintaining a Cu–S distance of 2.17–2.19 Å (DiDonato et al. 2000; Ralle et al. 2004; Walker et al. 2004). A similar Cu(I) coordination environment with a Cu–S distance of 2.16 Å was also shown to exist for MBDs of ATP7A (Ralle et al. 1998) and Atox1 (Ralle et al. 2003; Walker et al. 2004) via EXAFS measurements.

**Fig. 2:**
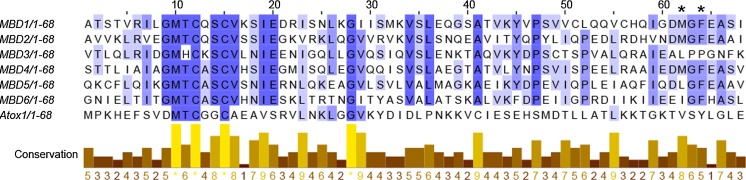
Multiple sequence alignment of individual ATP7B MBDs (MBD1-6) and Atox1. Residues that are highly conserved are highlighted with a dark purple color and color tone decays in parallel with the degree of conservation for that specific residue. Sequences between 3 and 70 for all MBDs and 1–68 for Atox1 were used. Sequence alignment was performed using Jalview version 2.10.2b1 (http://www.jalview.org/)

At first glance, all Cu-binding motifs of MBDs appear identical (Banci et al. 2009a; Walker et al. 2004). However, the sequence alignment of all MBDs (Fig. 2) indicates some positions within this motif may differ from one MBD to another with the highest conservation around the Cu-binding motif (GMX_1_CX_2_X_3_CV). Here, in most MBDs except MBD3, X_1_ position is occupied by threonine (T), whilst in MBD3, it is replaced by a histidine (H). Another substitution around the Cu motif is position X_2_ where a polar/uncharged glutamine (Q) in both MBD1 and MBD2 is changed to a lysine (K) in MBD3 and an alanine (A) in MBD4-6. Besides the Cu-binding motif, there is a second cluster of conserved residues which are located around phenylalanine (F66) (Fig. 2, F66 marked with asterisk). In silico, F66 was predicted to be in the α2-β4 loop in close proximity to Cu-binding motif (Rodriguez-Granillo et al. 2009). Here, a D_63_MGFEA_68_ motif is preserved for MBD1, MBD2 and MBD4. In MBD3, the motif is instead A_63_LPPGN_68_, and in MBD6 there are D63E, M64I and E67H substitutions. In MBD5, there is only one substitution (M64L) in this motif. The functional importance of these substitutions in individual MBDs is unclear but could be related to unique properties of individual domains (Bartee et al. 2009; Cater et al. 2004; Fatemi et al. 2010) and perhaps might help to explain why not all MBDs are functionally equivalent (Banci et al. 2009a; Cater et al. 2004; Iida et al. 1998; Rodriguez-Granillo et al. 2009; Wernimont et al. 2004).

In silico studies showed that the Cu-loop of most individual MBDs is stabilized by the hydrogen bonds between C_1_, the highly conserved threonine (T = X_1_) and serine (S = X_3_). The stabilization of the Cu-loop is strengthened by the methionine (M) residue preceding X_1_, which is fulfilled through the hydrophobic interaction between the M residue and the conserved phenylalanine (F66). C_2_ is buried in the core of the domains, and interacts with F66 or X_3_ (= S), M64 (Fig. 2, marked with asterisk) or with C_1_ to maintain a stable Cu-loop. C_2_ is buried deeper in MBD3 than in other domains, but does not interact with X_3_ or M64, instead of interacting with valine (V18) in helix α1 and V36/L38 in a β-strand. In contrast to other MBDs, MBD4 lacks most of the described interactions around the Cu loop due to the increased dynamics between α1-helix and last part of the Cu loop, which is absent in the other MBDs. The Cu-site in MBD4 is only stabilized through interactions of M with L40 and G43 that are located in the β2-β3 loop. C_1_ here interacts only with L40 but not with X_1_ (= T) or X_3_ (= S), and C_2_ of MBD4 is buried to the core and makes contacts with several residues including X_1_ (= T), X_3_ (= S), V20 in α1-helix, V38 in β2-strand and L40. Upon Cu-binding, C_1_ and C_2_ of MBD2 and MBD6 form hydrogen bonds with X_1_ (= T) and X_3_ (= S) and M became completely buried within the structure but like in the apo forms, continued to interact with residues in the β2–β3 loop, F66 and C_2_. The positioning of C_2_ in MBD6 becomes more buried than it is in MBD2 upon Cu binding, because it points toward the loop backbone. In MBD4, upon Cu-binding the interactions of M residue greatly varies but ultimately this residue points to the Cu-loop, contributing to the increased fluctuations of the Cu-loop. Moreover, Cu-bound C_2_ stops interacting with the F66 side chain and hence F66 forms contacts with the hydrophobic core. Although the Cu loop becomes more flexible in MBD4 upon Cu-binding, Cu-coordination was observed to reduce overall backbone fluctuations for all domains including MBD4 (Rodriguez-Granillo et al. 2009), which stabilizes the ferredoxin-fold of each domain (Portmann and Solioz 2005; Tadini-Buoninsegni et al. 2010).

### Long-range copper-induced effects in MBDs

Despite differences described above, all MBDs can bind Cu(I) with a similar binding affinity (Lutsenko et al. 1997; Yatsunyk and Rosenzweig 2007). However, isothermal titration calorimetry (ITC) experiments indicated that both MBD3–4 and MBD5–6 bind Cu-more tightly than MBD1–2 when they are studied as two-domain constructs but this distinction disappears when the domains are either accommodated in MBD1-6 (Wernimont et al. 2004; Yatsunyk and Rosenzweig 2007) or monitored separately in a two-domain construct (Banci et al. 2008). In silico studies with two-domain constructs revealed that the motional freedom of the Cu-binding loop undergoes a different behavior when covalently linked to a consecutive domain (Rodriguez-Granillo et al. 2010b). Although it has been proposed that progressive Cu binding induces conformational rearrangements along the overall N-terminal (DiDonato et al. 2000; DiDonato et al. 1997), NMR, CD and EXAFS studies performed on MBD1–6 construct (Banci et al. 2009a; Banci et al. 2009b; Banci et al. 2008) showed that Cu binding does not affect the overall fold of individual domains except for some changes localized only to the Cu-binding loop. These changes were proposed to be transmitted to other parts of the MBD1–6 construct through the peptide backbone (Banci et al. 2008; DiDonato et al. 2000). This suggestion agrees well with NMR and proteolysis results showing that Cu binding to MBD1–4 makes four domains to be organized in a more compact manner without triggering any major structural changes within each domain (Bartee et al. 2009; Mondol et al. 2016). However, NMR studies with MBD4-6 and MBD5-6 revealed that Cu-induced changes were only limited to the residues surrounding the Cu-binding motif and the rest of the protein structure remained largely unaffected (Achila et al. 2006; Fatemi et al. 2010). A recent in vitro study with MBD1-6 reported that MBD1-3 was compacted without Cu and adopted an extended conformation after Cu loading (Yu et al. 2017b), indicating a possible impact of Cu binding on the structural arrangements of both linkers and MBDs. Clearly, there should be more analyses to perform because constructs with different number of MBDs may behave differently.

Thermal unfolding experiments with MBD1–4 indicated that upon Cu-binding thermal midpoint (Tm) increased from 51 to 59 °C. This occurrence may be caused by the increased interactions between individual MBDs (Mondol et al. 2016). Similarly, Cu-binding to MBD5–6 construct increased Tm from 68 to 78 °C, which was reasoned that the binding of Cu to one domain increased the stability of both domains (Nilsson et al. 2013). Earlier an in silico study suggested that the interface region between MBD5 and MBD6 contains charged residues where R53 in MBD6 facing the protein surface interacts with F7 and E45 that are located in β1/β3 strands of MBD5. These interactions contributed to the stabilization of the entire construct (Rodriguez-Granillo et al. 2009). The NMR structure of MBD56 (PDB code: 2EW9) also shows that conserved K119 of MBD6 interacts with negatively charged residues (E73, E83 and D74) that are located either in the linker region or in strand β5 of MBD6. Due to these ionic interaction network, altered salt and pH conditions were found to dramatically affect MBD5-6 thermal stability (Nilsson et al. 2013). The deprotonation/protonation changes of histidine (H100 and H148) residues were proposed to affect the overall network of interactions on the protein’s surface (Nilsson et al. 2013). In addition, conformational changes within connecting linkers might modulate structural stability of the overall N-terminus (Bartee et al. 2009; DiDonato et al. 2000; Huster and Lutsenko 2003). These flexible linkers can also provide docking sites for the trafficking machinery (Hasan et al. 2012; Lim et al. 2006) and other interacting partners such as Atox1 and COMMD1 (Fatemi et al. 2010). The linker between MBD3 and MBD4 was shown with NMR, single-molecule fluorescence resonance energy transfer (smFRET) and proteolysis experiments to undergo conformational changes, such that it became more dynamic when the MBDs were loaded with Cu (Bartee et al. 2009; Fatemi et al. 2010; Keller et al. 2012). Taken all together, it is plausible that Cu coordination induces altered spatial organization of the six MBDs locally or globally, thereby modulating the overall protein activity.

### MBD interactions with other domains and proteins

**Fig. 3:**
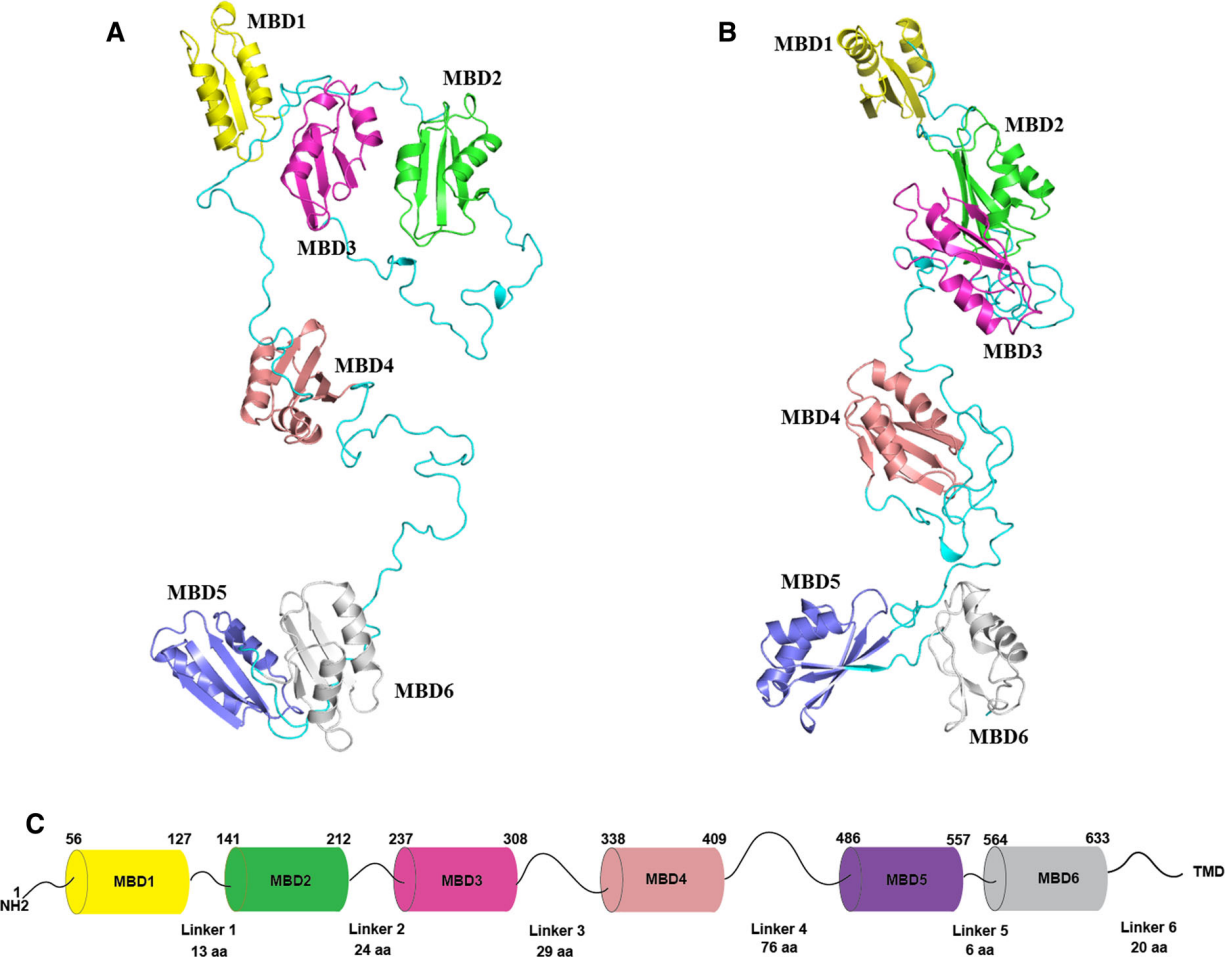
An all-atom full-length model of MBD1-6. **A** The initial model. **B** The optimized model. Six subdomains are colored differently, and their unstructured linkers are colored in cyan. NMR structures in Table 2 were used. The initial domain arrangement was determined based on the sequence length of linkers and electrostatic complementarity of the protein surfaces between different domains. 3D coordinates of the missing linkers were generated by SWISS-MODEL (Biasini et al. 2014) server using the arranged model as a template, leading to a full-length model of MBD1-6 without the inclusion of the first 56 residues. The initial model was then optimized by MD simulations using NAMD (version 2.12) (Phillips et al. 2005) for 5 nanoseconds (ns). The solvent was presented in the generalized Born/solvent-accessible surface area implicit solvent model (Tanner et al. 2011), and the protein system was described in CHARMM36 force field (Huang and MacKerell 2013). **C** The schematic representation of N-terminal MBDs of ATP7B in tandem. The length of each MBD and linkers (as the position of amino acids) in between is indicated according to the PDB structures of the respective MBDs (Table 2). A linker is defined as the segment between folded 70-aa MBDs except only for MBD6 which is 68-aa long. TMD represents the transmembrane part of ATP7B and the linker between MBD6 and transmembrane helix 1 (TMD1) is 20-aa long (Lorinczi et al. 2008)

High-resolution structures of homologs to ATP7B have been reported, including class 1B *Legionella pneumophila* CopA (LpCopA) (Gourdon et al. 2011), class IIA sarcoplasmic reticulum Ca^2+^-ATPase (SERCA1a) (Olesen et al. 2004), class IIC Na^+^, K^+^-ATPase (Morth et al. 2007; Ogawa et al. 2009; Shinoda et al. 2009) and class IIIA H^+^-ATPase (Abrahams et al. 1994; Stock et al. 1999), but in none of them the position of an MBD was resolved. Although Gourdon et al. have proposed a full-length model of ATP7B using homology modeling (Gourdon et al. 2012) where MBDs were arranged as ‘stack of logs’, the true orientation of the MBDs is still unknown. To visualize the organization of the six MBDs at the atomic level, we developed a full-length model of the six MBDs by integrating current structural and biophysical data (Dmitriev et al. 2016; Yu et al. 2017a). The detailed information for building the model is described in Fig. 3. The initial model (Fig. 3A) was then sampled by MD simulations in implicit solvent to optimize the added linkers between different domains. In the resulting model (Fig. 3B), MBD1–3 and MBD5–6 act as two independent structural units and the orientation of MBD1-3 agrees with the data from recent small-angle X-ray scattering (SAXS) experiments (Yu et al. 2017b). By contrast, MBD4 undergoes few contacts with other domains in the model, in line with its proposed role in merely connecting other structural units (Yu et al. 2017b). Multi-scale modeling that integrates all-atom and coarse-gained models may provide a strategy to simulate a full-length ATP7B model (including MBDs, membrane-spanning parts, lipids etc.) in the future.

An interaction of MBDs with another ATP7B domain was first reported upon co-purification of MBDs with the N-domain. The interaction was found to depend on Cu such that it was detected only in the absence of Cu in the MBDs (Tsivkovskii et al. 2001). This interaction was proposed to keep ATP7B inactive at low Cu-conditions (Bartee et al. 2009; Tsivkovskii et al. 2001). Upon Cu-binding to MBDs, the structural reorganization resulted in dissociation of the MBD-N-domain complex and thereby allowed ATP to bind to the N-domain (Bartee et al. 2009). However, NMR studies with MBD1–4 and MBD1–6 constructs could not detect any stable interaction with the N-domain in solution although very high concentrations were used. Mondol et al. therefore suggested that the N-domain-MBD complex formed only transiently, or constituted a minor species in the co-purification experiments (Mondol et al. 2016). The homology model of ATP7B, based on the LpCopA, also indicated that MBD6, with an overall positive surface charge, may interact with the negatively charged P-domain (Gourdon et al. 2012) and N-domain (Dmitriev et al. 2006). As the latest piece of information, a synthetic peptide comprising residues 33–63 of ATP7B (i.e., peptide stretch just before MBD1) was found by NMR to interact at the interface between N- and A-domains despite the usage of a high concentration of the peptide (Yu et al. 2017b). More investigations are needed to understand how the N-terminal MBDs interact with the rest of the ATPase cytoplasmic domains, and the resulting consequences for Cu transport function.

### Atox1 interactions with MBDs

Prokaryotic ATPases from different organisms can contain up to four MBDs and many of them sequester its metal directly from cytoplasm where they do not strictly require a cytoplasmic chaperone (Sharma and Rosato 2009). In humans, however direct protein–protein interactions between Atox1 and MBDs are essential for the delivery of Cu to the MBDs (Hamza et al. 1999; Hussain et al. 2009; Larin et al. 1999; Walker et al. 2002, 2004; Yu et al. 2017b). After the initial Cu-transfer from the chaperone, Cu is most likely channeled through MBDs according to the proposed “bucket-brigade” model where Cu is moved along MBDs (Bunce et al. 2006; Walker et al. 2004) and later delivered to MBD6, which is believed to forward Cu ions to the intramembrane Cu-sites (CIA**CPC** motif in TMD6, Fig. 1). An inter-MBD Cu-transfer reaction was shown in vitro by NMR to take place from MBD4 to MBD6 in a construct containing MBD4-6 (Achila et al. 2006). ITC data revealed that affinities of MBDs for Cu was very similar among six domains and to that of Atox1 (Benitez et al. 2011) suggesting that Cu-exchange between Atox1 and MBDs, and among MBDs, may be under kinetic rather than thermodynamic control (Wernimont et al. 2004; Yatsunyk and Rosenzweig 2007).

Atox1 can deliver Cu (I) to all MBDs in vitro (Banci et al. 2008; Walker et al. 2002; Yatsunyk and Rosenzweig 2007) but not all MBDs are equivalent in terms of receiving Cu from Atox1 (Achila et al. 2006; Fatemi et al. 2010). Titration of apo-MBD2 (Walker et al. 2004), apo-MBD4 and apo-MBD5–6 (Achila et al. 2006) with Atox1-Cu complexes revealed that Atox1 delivers copper specifically to MBD2 and MBD4 forming an adduct with the chaperone whereas MBD56 is partially metallated without complex formation in accordance with the findings of a two-hybrid assay (Banci et al. 2009a; Larin et al. 1999). In fact, only MBD1, MBD2 and MBD4 were demonstrated to form Cu(I)-bridged adducts with Atox1, whereas no adduct formation takes place for MBD3, MBD5 and MBD6 but there is still Atox1-mediated Cu transport (Banci et al. 2008; Bunce et al. 2006). The electrostatic potential surface of Atox1 is dominated by the positive potential due to the presence of arginine (R) and lysine (L) residues in the helix α1 and helix α2. However, the surface charge distribution varies in different MBD domains; MBD1 (the least negative MBD), MBD2, MBD4 and MBD5 contain more negatively charged patches while MBD3 and MBD6 have more positively charged surfaces. MBD4 is the most negative one among the six domains, suggesting that this domain contributes more than others to the interaction between the positively charged Atox1 and negatively-charged MBDs (Rodriguez-Granillo et al. 2009). In accordance with this, NMR studies with MBD2 and MBD4 indicated that both MBD2 and MBD4 interact similarly with Atox1 (through similar residues) where positively charged residues in Atox1; R21, K25, K56 and K57 showed significant chemical shifts in the presence of these MBDs (Achila et al. 2006) indicating a possible ionic interaction network at the interface. A mutational study also showed that K60 of Atox1 is a critical position for complex formation with MBDs so that K60A/K60Y Atox1 mutations disrupted MBD4 binding (Hussain et al. 2009). Taken together, it could be stated that electrostatic interactions between these positively charged residues in Atox1 and negatively charged residues at the surface of MBDs complement each other, facilitating Cu-dependent interaction and thereby Cu-transfer (Bunce et al. 2006; Hamza et al. 1999).

MD calculations showed that Atox1 binds to MBD4 stronger than to any other MBDs (Rodriguez-Granillo et al. 2009; van Dongen et al. 2004). In the Atox1-MBD4 complex, Cu bridges the proteins by coordinating C residues of both proteins (Banci et al. 2009a; Benitez et al. 2011; Larin et al. 1999; Lutsenko et al. 1997). In silico, it has been shown that 2-coordinated intermediates are not likely to occur during Atox1-Cu-MBD4 complex formation and a stable adduct is formed with a 3-coordinated intermediate geometry that is; Cu is coordinated via two cysteines from one protein and one cysteine from the other. Such adduct is considered as a more stable geometry than 2- or 4-coordinated counterparts (Rodriguez-Granillo et al. 2010a), which is supported by the experimental findings with MBD4 (Niemiec et al. 2015) and MBD1–6 (Ralle et al. 2004). Using a combination of SEC and ITC experiments performed on MBD4 and Atox1, Niemiec et al. showed that hetero-protein complex formation occur via two steps; reactants to hetero-protein intermediate (step 1) and intermediate complex to products (step 2) (Niemiec et al. 2012) where the first step involves large favorable enthalpy and entropy changes (Niemiec et al. 2015). In contrast to the bulk solution results, smFRET experiments showed that apo-forms of Atox1 and MBD4 interact with each other, but Cu-bridging was shown to further stabilize the protein complex (Benitez et al. 2011).

### WD-causing mutations in MBDs of ATP7B

WD is caused by mutations which can impede every step of the catalytic cycle of ATP7B and the final impact on the protein can range from mild to severe depending on which residues are affected (Huster et al. 2012). WD disease-causing mutations can be missense, frameshift, non-sense or splice-site mutations (Harada et al. 2001), however a large of number of reported WD mutations are missense (Chen et al. 2015; Huster et al. 2012). Most mutations observed in patients tend to be clustered in conserved regions of the ATPase core especially in the transmembrane region (Forbes and Cox 1998; Huster et al. 2012; Schushan et al. 2012) but some are also observed at other parts of the ATPase structure (e.g. A-domain, MBDs). The three most common mutations are as follows; R778L (located in TMD4 close to the A-domain) is the most common mutation worldwide (58% of all WD cases) and observed in patients of southeastern Asian descent (mostly Korean and Chinese patients), the second most common mutation H1069Q (found in N-domain; 35–45% of all mutations) is prevalent among European descent (Europe and North America) and is followed by R778W mutation which is prevalent among Indian population (Hedera 2017; Inesi et al. 2014; Jang et al. 2017).

**Fig. 4:**
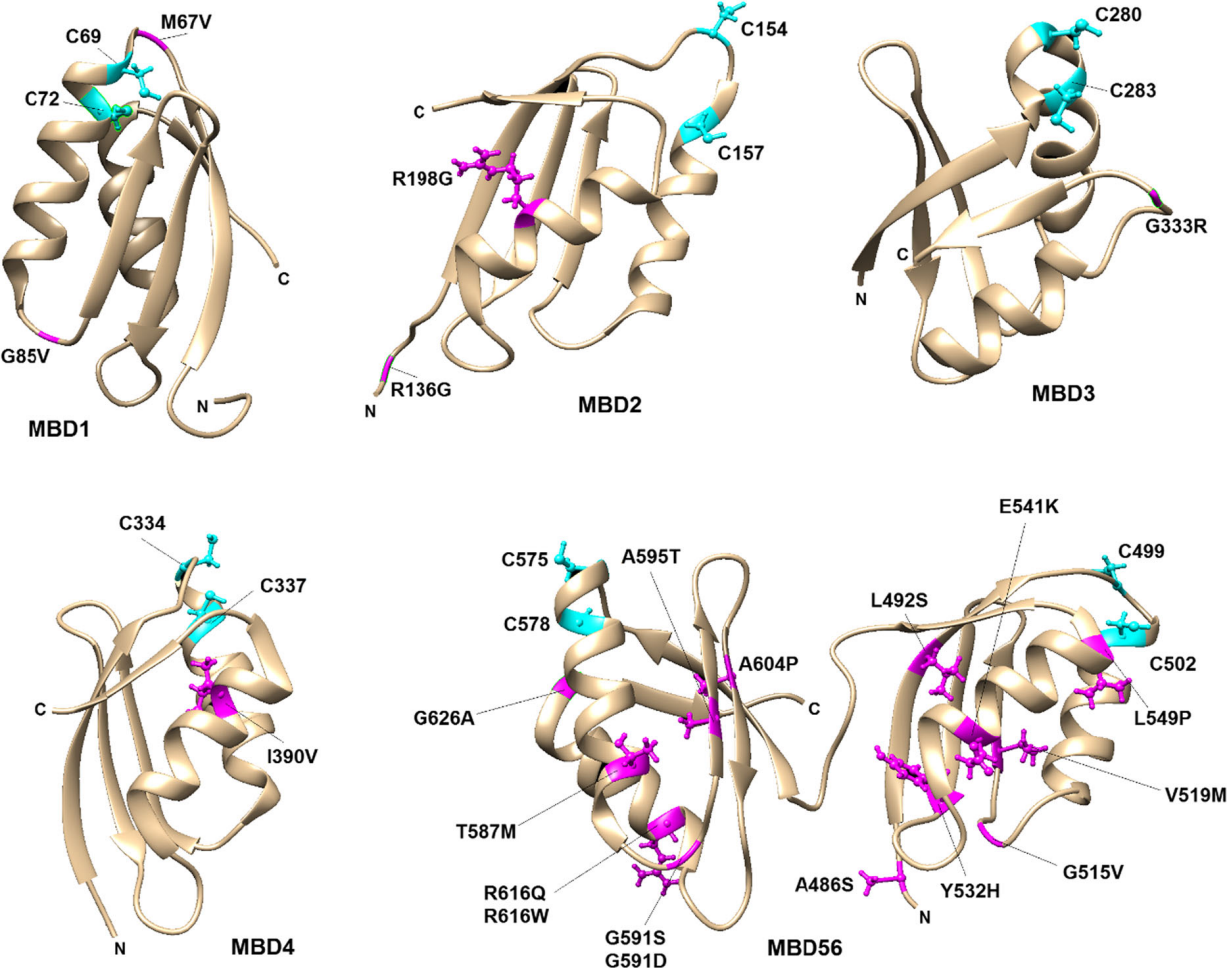
The positioning of WD-causing mutations (Table 1) in each MBD. The disease-causing mutations were visualized in magenta using UCSF Chimera molecular-modeling software. For all MBDs Cu-binding cysteines (C1 and C2) are shown with ball-stick representation in cyan. Mutations N41S in MBD1 and S406A, V456L, A476T in MBD5 could not be represented here due to the absence of these positions in reported PDB structures

To date, more than 800 mutations are reported with observed clinical symptoms (**H**uman **G**ene **M**utation **D**atabase, HGMD; http://www.hgmd.org) but the wide-spectrum of phenotypic variations of these mutations often makes it hard to make an accurate and early clinical diagnose for WD (Braiterman et al. 2014; Dong et al. 2016; Jang et al. 2017). Due to a wide variety of symptoms and age of onset, knowledge of how mutations in ATP7B link to symptoms in patients is still inconclusive. A number of studies, both biological/biochemical and biophysical, characterized WD-causing mutations which showed that these mutations may have various effects on ATP7B function (Braiterman et al. 2014; Chen et al. 2015). The most commonly observed effect is “protein misfolding” where ATP7B is retained in endoplasmic reticulum (ER) with a marked decrease in protein stability and thus results in the loss of Cu-transport in cells (Concilli et al. 2016; Payne et al. 1998; Tsivkovskii et al. 2003). Moreover, other effects such as impaired protein–protein interactions, decreased/increased phosphorylation state, altered binding affinities for ATP or Cu, altered conformational changes or cellular localization/vesicle trafficking behavior (Chen et al. 2015; Huster et al. 2012) have also been reported. We here gathered the known WD missense mutations localized in the six MBDs (Table 1). It is notable that WD-causing mutations in every MBD has been reported, but MBD5 and MBD6 contain two thirds of reported mutations. This is likely due to their location close to the Cu entry site in the membrane part of ATP7B and hints to key roles of this region in ATP7B function. The majority of the mutated positions are part of α-helices (e.g., R198G, I390V, E541K, L549P, T587M and R616Q etc.), linker regions (e.g., M67V, R136G, G515V and G591S etc.), whereas a few reside in β-strands (e.g., V519M and A595T etc.) (Fig. 4). Most of the WD mutations in Table 1 lack in vitro or in vivo information for how they affect ATP7B function, but below follows what has been reported.

G85V (in MBD1) and L492S (in MBD5) ATP7B variants were reported to result in complete loss of catalytic Cu transport activity (Huster et al. 2012; van den Berghe et al. 2009), whereas S406A, Y532H and G626A ATP7B variants were shown to maintain a normal Cu-transport activity in cell assays. G85, L492 and Y532 positions are distant to the Cu-site, and the mutations may thus affect domain–domain interactions or intrinsic structural stability. On the other hand, G626 is situated near the CxxC motif in MBD6 and MD simulations showed that G626A alters the hydrogen-bonding network thereby affecting Cu-binding ability (Hsi et al. 2008). We previously reported that G386V and G386D mutants (equivalent to G85 V and G591D but incorporated in MBD4) displayed dramatically reduced thermal stability compared to wild-type MBD4. Complementary MD simulations showed that the mutated residues enhance overall domain fluctuations (Kumar et al. 2017) which may alter how MBDs interact with Atox1, other MBDs, or other interacting partners. In accordance with this, ATP7B with the G85V mutant was reported to have a decreased interaction with Atox1 in cell-based assays. This was also found for G591D and L492S variants of full-length ATP7B when using GST-pulldown analysis (Hamza et al. 1999; Pilankatta et al. 2011). All three mutations showed increased interactions with MURR1-containing domain 1 (COMMD1), a scaffold protein responsible for protein ubiquitination and proteosomal degradation (de Bie et al. 2007; Hamza et al. 1999; Vonk et al. 2014). G85V ATP7B was observed in cell studies to mislocalize to the ER along with reduced protein expression (van den Berghe et al. 2009). In contrast, the G591D (Hamza et al. 1999; Pilankatta et al. 2011; Vanderwerf et al. 2001) and L492S (Hamza et al. 1999) variants were shown to traffic normally, but L492S was reported to be defective in Cu transport (Huster et al. 2012) whereas G591D resulted in impaired Cu-mediated ATPase activity (Vanderwerf et al. 2001). Characterization of additional WD mutations in individual domains and in full-length ATP7B using in vitro and in vivo approaches may contribute to further knowledge of underlying mechanisms.

## Outlook

In Atox1-ATP7B pathway, Cu ions are believed to be transferred from Atox1 to some or all MBDs, then forwarded to the Cu-site in the transmembrane channel and finally delivered to Cu-dependent proteins passing the lumen of the Golgi. To better understand how the MBDs modulate ATP7B function, a high-resolution X-ray structure of full-length ATP7B would be extremely helpful but appears to be difficult to obtain as there are many different arrangements of the different domains in ATP7B. Instead, cryo-EM may be a promising approach for information on the spatial organization of the MBDs in ATP7B as a function of conditions. In the bigger picture, Cu-transfer mechanisms is of great importance to identify since Cu-transport dysregulation has been reported for several cancers (e.g., lymphoma, reticulum cell sarcoma, bronchogenic and laryngeal squamous cell carcinomas, cervical, breast, stomach and lung cancers) (Denoyer et al. 2015) and in some neurodegenerative diseases (Alzheimer’s disease, amyotrophic lateral sclerosis, Huntington’s disease, Parkinson’s disease, and prion diseases etc.) (Chen et al. 2016). In fact, in some cancers ATP7B expression has been connected to tumor-cell differentiation and alteration of outcome for platinum-based chemotherapy drugs (Dmitriev 2011; Kuo et al. 2007; Li et al. 2014; Martinez-Balibrea et al. 2009; Yang et al. 2015). For instance, cisplatin, a potent anti-cancer agent used against solid tumors of various cancers has been shown to bind to both Atox1 and ATP7B, perhaps processes governing cell resistance against this drug (Dmitriev 2011; Palm-Espling et al. 2013; Palm et al. 2011). Despite a lot of excellent biophysical work, further studies on ATP7B mechanisms, and the roles of the MBDs, are desired. This will aid not only better understanding of normal Cu transport and development of WD, but also the role of Cu in cancer and neurodegeneration processes.

## Electronic supplementary material

Below is the link to the electronic supplementary material.

Supplementary material 1 (PDF 125 kb)
