# Supplementary material for: The six metal binding domains in human copper transporter, ATP7B: molecular biophysics and disease-causing mutations
Source: Biometals. 2017 Oct 23;30(6):823–40. doi: 10.1007/s10534-017-0058-2 (PMC5684295; doi:10.1007/s10534-017-0058-2)
Supplement: Supplementary file 1 — Supplementary material 1 (PDF 125 kb) [file 10534_2017_58_MOESM1_ESM.pdf]

Biometals

Supplementary Information

The six metal binding domains in human copper transporter, ATP7B: Molecular biophysics and disease-causing mutations

Candan Ariöz, Yaozong Li, Pernilla Wittung-Stafshede*

Table S1. Summary of studies reporting biophysical data on MBD1-6 in the literature.

Construct	Analysis method		Biophysical outcome	Ref
MBD2	In vitro	Fluorescent detection/trypsin digestion/N-terminal amino acid sequencing	MBD2 is very similar to other MBDs for its domain exposure or affinity towards Cu. Specific protein-protein interactions with Atox1 are essential for delivery of copper to the preferential site.	1
		Fluorescence Cu-transfer assays (Cu^+-Atox1 or free Cu^+)	Cu-binding to MBD2 results in conformational reorganization within MBDs allowing Atox1 to access sufficiently to other MBDs.	
		Competition experiments using absorption spectroscopy	The affinity of MBD2 for Cu was significantly higher than that of Atox1.	
		Radioactive phosphorylation assay using γ-[^{32}P] ATP and MBD2 (C→A mutant)	MBD2 is essential for the initial steps of Cu-transfer from Atox1 to the catalytically essential intramembrane Cu-binding sites.	
MBD4		Single-molecule fluorescence resonance energy transfer (smFRET)/ nanovesicle trapping scheme	The stability of MBD4-HAH1 interaction complex depends on Cu^{1+} bridging at the protein interface.	2
		Thermal denaturation experiments/far-UV CD spectroscopy using MBD4 and G386V/G386D mutants	G386V/G386D resulted in reduced MBD4 thermal stability and Cu-binding further promoted instability.	3
	In silico	Molecular dynamic (MD) simulations of MBD4/G386V/G386D mutants	Mutations increased backbone fluctuations that extended throughout the domain and resulted in increased structural dynamics. G386V underwent largescale conformational changes which leads to a transition to a β-sheet-rich structure.	
		MD simulations of MBD4-Atox1 interactions	MBD4 form stable adducts with Atox1 mutants, M10A and T11A. No heterocomplex formation observed between MBD4 and K60A/K60Y Atox1 variants which caused MBD4's Cys to be more distant from holo-Atox1's Cu site.	4
	In vitro	Near-UV CD and gel filtration combined with ICP-MS		
		Size exclusion chromatography (SEC)/titration calorimetry using (C→A) mutants of Atox1 and MBD4	Only two tricoordinate Atox1-Cu-WD4 complexes form *in vitro*. Cu-dependent complexes require the presence of C1 but not C2 for a successful Cu transfer.	5
		Isothermal titration calorimetry (ITC) experiments	In MBD4/Atox1 reaction, a strong entropy-enthalpy compensation is in charge both at Step 1 (reactants to heterocomplex) and Step 2 (heterocomplex to products).	
		SEC/ITC	Step 1 corresponds to a ΔG change of –30 kJ/mol and step 2 of +25 kJ/mol; resulting in an overall driving force for vectorial transfer towards MBD4 of –5 kJ/mol.	6
		Isothermal titration calorimetry (ITC)	Hetero-complex formation between Atox1 and MBD4 involves negative enthalpy and positive entropy changes. MBD4 binds Cu with a ~5 kJ/mol lower free energy (higher affinity) than Atox1.	
	In silico	MD simulations of MBD4-Atox1 interactions	During the Cu (I)-transfer process from Atox1 to MBD4, 2-coordinated intermediates are not likely to occur. 3-coordinated intermediates are more stable than 2-coordinate or 4-coordinate intermediates.	7
		Quantum mechanics/molecular mechanics (QM/MM) calculations	Cu-transfer reaction from Atox1 to MBD4 appears to be kinetically accessible but less energetically favorable (ΔE) 7.7 kcal/mol).	
	In vitro	SEC/ITC	T at the X_1 position in MBDs of ATP7B have been conserved to facilitate the directional transfer of Cu from Atox1 to MBDs (here MBD4). T in the X_1 position destabilizes Atox1-Cu-WD4 hetero-complex forcing Cu to the MBDs, whereas a D at the same position have a stabilizing effect on the hetero-complex making the metal (Zn) to move toward the Atox1.	8
MBD1/ MBD4		Copper transfer assay using gel-filtration chromatography/ICP-MS	Irrespective of the copper donor, copper transfer from MBD1 to MBD4 or vice versa occur. Domain-domain communication exists between MBDs.	9

MBD3/ MBD4	In vitro	smFRET/vesicle trapping	HAH1 interaction with double-domain construct is more stable than its interaction with single domain constructs. HAH1-MBD3 and 4 interactions showed similar stabilities. Excess Cu^{1+} decreased the stabilities/lifetimes of HAH1-MBD3/MBD4 complexes.	10
	In silico	Thermodynamic analyses/MD simulations of smFRET data	The dynamic linker between MBD4 and MBD3 may also play a role in the complex formation.	
MBD2/ MBD4	In vitro	NMR Titrations of MBD2 and MBD4 with Cu-HAH1 complex	HAH1 interacts similarly with MBD2 and MBD4 (through similar residues) but interaction between HAH1 and MBD4 is significantly stronger than the interaction with MBD2.	11
MBD4/ MBD5-6		NMR titrations of MBD5-6 with Cu-MBD4 and Cu-HAH1 complexes	Cu-transfer from HAH1-Cu to MBD5-6 is impaired. Partial Cu-transfer from MBD4-Cu to only MBD6 (1:1 ratio) occur while Cu-MBD4: MBD5-6 ratio increases up to ratio 6:1, MBD5 and MBD6 are both metallated. MBD5-6 function as a single unit but acquire copper differently because of the location of metal-binding motifs on opposite ends of the molecule.	
MBD3-4		NMR spectroscopy	MBD3 and MBD4 both interact with the same domain of another MBD3-4 molecule. Cu (I)-HAH1 forms detectable amounts of stable adducts with MBD4, whereas MBD3 removes Cu (I) from HAH1 without detectable complex formation.	12
MBD4-6		NMR spectroscopy	The linker joining MBD4 to MBD5-6 retains its flexibility in both the apo and Cu (I)-bound states and becomes even more mobile in the Cu (I)-bound state. MBD4 is the site for Cu-delivery to Atox1 and have a higher motional freedom than MBD5 and MBD6.	13
		NMR relaxation experiments	MBD5 and MB6 have flexibility with respect to each other eventhough they have a short linker. Relative orientations and mobilities of the domains in MBD4-6 do not change significantly upon binding copper (No conformational change due to Cu-binding).	
MBD5-6		Far UV-CD spectroscopy/thermal denaturation experiments	Thermal stability of MBD5-6 is pH-dependent.	14
		NMR spectroscopy/relaxation experiments	The second Cys residues in each Cu site and His residues (H100 and H148) that are in close proximity to Cu site (in 3D structure) are protonated with lower pH (6.0) but deprotonated at high pH (7.2).	
	In silico	MD simulations	Inter-domain interactions fluctuate more and protein becomes more susceptible to temperature increase at low pH and high salt conditions than it is at high pH and low salt condition.	
	In vitro	Cu-loading experiments with size-exclusion (SEC) chromatography	Atox1 transfer of Cu to MBD56 is more favorable at high pH (pH 7.2; Kex = 15) than at low pH (pH 6.0; Kex = 0.5) by a factor of 30. Cu-transfer is also pH dependent.	
MBD1-4		Far UV-CD spectroscopy	Cu-binding to MBD1-4 increased thermal stability and also induced conformational changes of residues distant from Cu-binding sites resulting in compaction of four individual metal binding domains.	15
		NMR spectroscopy/diffusion experiments	Compaction of MBD1-4 with Cu-binding abolishes transient interactions between MBD1-4 and N-ATP7B.	
MBD1-6		Immobilized Metal Ion Affinity Chromatography (IMAC)	The affinity of ATP7B for different metals were highest for Cu (II) and is followed by Zn (II), Ni (II), Co (II).	16
		Competition blotting experiments using ^{65}Zn (II)	The affinity of MBDs-ATP7B domain for Cu increases with increasing amount of Cu (I or II) so that Cu-binding by the domain is to some degree cooperative.	

MBD1-6	In vitro	Far UV-CD spectroscopy	Progressive Cu-binding to MBD1-6 induce structural changes (both in the secondary and tertiary structure) in the domain.	17
		X-ray Absorption Spectroscopic (XAS) analysis	All Cu-binding sites bind Cu with a distorted diagonal geometry and this geometry does not change with different Cu:protein stoichiometries.	
		Copper transfer experiments using an amylose resin	Atox1 can deliver copper to all metal-binding sites in MBDs-ATP7B in a dose-dependent and saturable manner.	18
		Radioactive phosphorylation assay using γ-[^{32}P]ATP	Cu-Atox1 transfers copper to the ATP7B metal binding sites, which are essential for the stimulation of its catalytic phosphorylation.	
		Fluorescent detection with CPM-labeling	Reverse Cu-transfer is partial and MBD1-6 cysteines remains reduced after the reverse Cu-transfer.	
		Radioactive phosphorylation assay using γ-[^{32}P]ATP	apo-Atox1 leads to a concentration-dependent and saturable reduction of the ATP7B activity (downregulation of enzymatic activity but not total inhibition).	
		Solution NMR spectroscopy	HAH1 forms Cu (I)-bridged adduct with MBD1, MBD2 and MBD4 in a Cu-dependent manner. MBD3, MBD5 and MBD6 have a metal transfer directly from HAH1 without the adduct formation.	19
		NMR spectroscopy	MBD1-4 interact with HAH1 more strongly than MBD1-6.	20
		Column binding assays using fusion proteins with maltose-binding protein (MBP)	MBD1-6 binds specifically to HAH1 protein and this interaction requires specifically Cu as a metal and a quadruple coordination of Cu between MBD1-6 and HAH1. Interaction of Cu-loaded MBD1-6 with HAH1 is reversible.	
		Metabolic-labeling studies using orthophosphate (^{32}P)	ATP7B$^{R875,S340/341G}$ had %30 lower phosphorylation than ATP7B^{R875} mutant and presence of Cu increased the difference in phosphorylation levels observed for both mutants.	21
		NMR Analysis	S340/341A mutation does not significantly affect protein folding of MBDs-ATP7B.	
		Co-purification experiments using fusion proteins with maltose-binding protein (MBP)	S340/341A mutant showed a ~50% decrease in inter-domain interaction between MBDs and N-ATP7B. Ser-340/341 influences the interdomain interface and overall conformation of ATP7B.	
		Fluorescence spectroscopy	MBD2 C→A mutant showed a conformational change with a greater exposure of Trp in the loop connecting MBD1 and MBD2 to solution whereas, MBD2 C→S mutant had no change in Trp fluorescence. This indicates that compact packing of MBDs result in conformational changes that expose loops that are inaccessible.	22
	In silico	MD simulations	In the apo form, the metal-binding loop of MBD2 is flexible enough to reach and interact with other MBDs and that the ability of sulfhydryl groups to hydrogen bond is important for the inter-domain interactions.	
			Cu (I) binding to one MBD induced changes not only in individual domains but resulted in tertiary arrangements of the entire construct. Cu-binding induced a more compact tertiary structure, stabilizing MBD-MBD interactions with larger surface area.	23
	In vitro	X-ray absorption spectroscopy using MBP fusions	Cu (I) centers in MBD1-6-MBP fusion protein show a mixture of linear 2-coordinate and 3-coordinate geometries among all MBDS but majority of sites appear to be in a 2-coordinate state (Bis-cysteinate geometry).	24
	In silico	Molecular dynamics (MD) simulations	MBD4-Atox1 interaction is the strongest and occur via electrostatic interactions. Cu-binding reduces structural flexibility of all domains except MBD4.	25

MBD1-6	In vitro	The near/far-UV circular dichroism (CD) spectroscopy	N-terminal of the rat ATP7B protein (rCBD) retains its typical ferrodoxin fold of metal binding domains. Structural changes occur both in the secondary and tertiary structure of rCBD with Cu binding.	26
		^{65}Zn(II)-competition blotting	Cooperative interaction and high affinity of rCBD towards Cu.	
	In silico	Homology modelling of rCBD	rCBD have six metal binding domains but only five of MBDs contain CXXC Cu-binding motif. rD4 (4th metal binding domain) is missing the CXXC motif which is proven not to have a significant role in cooperative binding, structural/conformational changes and overall protein folding.	
	In vitro	Isothermal titration calorimetry (ITC) of MBD1-2, MBD3-4, MBD5-6, MBD1-4 and MBD1-6 domain constructs	The association constants are ~10^5 to 10^6 M^{-1} and are similar for Atox1 and target domains. The affinities for Cu are $MBD14_2$ >MBDD34 ≥MBD56 ≈ $MBD14_1$> MBD12 ≈ MBD16 ≈ Atox1. Copper handling by Atox1 and copper exchange between Atox1 and MBDs are under kinetic rather than thermodynamic control.	27
		β-galactosidase assay	The highest β-galactosidase activity for single copper-binding domains is measured for the interaction between ATOX1 and MBD4 of ATP7B. Protein-protein interactions is copper dependent, but is disrupted at high copper concentrations.	28
	In vitro	Copper transfer experiments using SEC	Atox1 can deliver Cu (I) to all of the MBDs *in vitro* through direct protein-protein interactions. Reverse Cu transfer is also possible but with lower efficiency 5-fold excess of Atox1 is required to remove Cu(I) from the MBDs.	29
		BCA competition assay	MBDs bind Cu (I) more strongly than Atox1.	
		Isothermal Titration Calorimetry (ITC)	Cu (I) binds to both Atox1 and the MBD1–6 variant proteins with similar binding constants (K_{Cu}) of 2.2-6.3x 10^{10} M^{-1}.	
		Circular dichroism (CD) spectroscopy	Zinc binding to MBDs destabilizes the protein structure (both secondary and tertiary) but Cu binding have a stabilizing effect on MBDs.	30
		X-ray absorption spectroscopy (XAS)	Zinc atom coordinates with nitrogen ligands (Histidines)/a combination of nitrogen+sulfur (Histidine and cysteines) ligands when it binds to MBDs but Cu binds to MBDs with a distorted linear geometry using two sulfur ligands (cysteines).	
		ATPase assay	Metal ion specificity is determined by the transmembrane part of the ATPase and N-terminal cannot override the intrinsic specificity	31
		NMR spectroscopy	Interactions between MBD1 and MBD2 are insignificant and that, in the absence of the nanobody, both MBD2 and MBD1interact directly with MBD3. Unlike MBD2 and MBD3, MBD4 moves mostly independently and does not influence copper-binding characteristics of other MBDs.	32
		Nanobody detection with confocal microscopy	Nanobody-induced changes in the MBDs-ATP7B structure or dynamics modulate the trafficking response of ATP7B.	
		Metal-chelate chromatography on IAA (iminodiacetic acid agarose) resin	MBD1-6 specifically bind copper *in vitro,* when copper is added to this protein in a partially chelated Cu (II) form but copper bound to MBD1-6 is likely in Cu (I) form.	33
		CPM labeling/gel-based fluorescence detection	Cysteines are directly involved in the coordination of copper.<br>5–6 copper molecules are bound per N-terminal domain molecule.	
	In silico	Bioinformatic sequence analysis	Prokaryotic ATPases contain up to four MBDs. For a large number organisms, the ATPase does not strictly need to receive its metal substrate through a cytosolic chaperone but can directly sequester it in the cytoplasm.	34
		MD simulations	Both for apo and holo forms, MBD5 and MBD6 display increasing freedom of relative reorientation with increasing linker length and, in parallel, less significant energies of inter-domain interaction. MBD pairs closest to the transmembrane domain do not need to have a fixed orientation with respect to one another. Relatively short linker lengths already permit an appreciable degree of relative conformational freedom.	

Abbreviations:

* MBD: Metal binding domain of ATP7B

* MBD1-6: Metal binding domains 1, 2, 3, 4, 5 and 6 of ATP7B in tandem

* MBD5-6: Metal binding domains of 5 and 6 of ATP7B in tandem

* HAH1: Also known as Atox1

* MBDs-ATP7B: N-terminal of ATP7B

* ATP7B: Full-length ATP7B

References

1- Walker JM, Huster D, Ralle M, Morgan CT, Blackburn NJ, Lutsenko S (2004) The N-terminal metal-binding site 2 of the Wilson's Disease protein plays a key role in the transfer of copper from Atox1. J Biol Chem 279 (15): 15376-15384.
2- Benitez JJ, Keller AM, Huffman DL, Yatsunyk LA, Rosenzweig AC, Chen P (2011) Relating dynamic protein interactions of metallochaperones with metal transfer at the single-molecule level. Faraday Discuss 148: 71-82.
3- Kumar R, Ariöz C, Li Y, Bosaeus N, Rocha S, Wittung-Stafshede P (2017) Disease-causing point-mutations in metal-binding domains of Wilson disease protein decrease stability and increase structural dynamics Biometals 30: 27-35.
4- Hussain F, Rodriguez-Granillo A, Wittung-Stafshede P (2009) Lysine-60 in copper chaperone Atox1 plays an essential role in adduct formation with a target Wilson disease domain. J Am Chem Soc 131 (45): 16371-16373.
5- Niemiec MS, Dingeldein APG, Wittung-Stafshede P (2015) Enthalpy-entropy compensation at play in human copper ion transfer. Sci Rep **5**: 10518. doi: 10.1038/srep10518
6- Niemiec MS, Weise CF, Wittung-Stafshede P (2012) In vitro thermodynamic dissection of human copper transfer from chaperone to target protein. PloS ONE 7 (5): e36102.
7- Rodriguez-Granillo A, Crespo A, Estrin DA, Wittung-Stafshede P (2010) Copper-transfer mechanism from the human chaperone Atox1 to a metal-binding domain of Wilson Disease protein. J Phys Chem B 114: 3698-3706.
8- Niemiec MS, Dingeldein APG, Wittung-Stafshede P (2014) T versus D in the MTCXXC motif of copper transport proteins plays a role in directional metal transport. J Biol Inorg Chem 19: 1037-1047: doi 10.1007/s00775-014-1147-0
9- Bunce J, Achila D, Hetrick E, Lesley L, Huffman DL (2006) Copper transfer studies between the N-terminal copper binding domains one and four of human Wilson protein. Biochim Biophys Acta 1760: 907-912.
10- Keller AM, Benitez JJ, Klarin D, Zhong L, Goldfogel M, Yang F, Chen TY, Chen P (2012) Dynamic multibody protein interactions suggest versatile pathways for copper trafficking. J. Am. Chem. Soc. 2012, 134, 8934−8943.
11- Achila D, Banci L, Bertini I, Bunce J, Ciofi-Baffoni S, Huffman DL (2006) Structure of human Wilson protein domains 5 and 6 and their interplay with domain 4 and the copper chaperone HAH1 in copper uptake. PNAS 103 (15): 5729-5734.
12- Banci L, Bertini I, Cantini F, Rosenzweig AC, Yatsunyk LA (2008) Metal binding domains 3 and 4 of the Wilson Disease protein: Solution structure and interaction with the copper (I) chaperone HAH1. Biochemistry 47: 7423-7429.
13- Fatemi N, Korzhnev DM, Velyvis A, Sarkar B, Forman-Kay JD (2010) NMR Characterization of copper-binding domains 4-6 of ATP7B. Biochemistry 49: 8468-8477. doi: 10.1021/bi1008535
14- Nilsson L, Ådén J, Niemiec MS, Nam K, Wittung-Stafshede P (2013) Small pH and salt variations radically alter the thermal stability of metal-binding domains in the copper transporter, Wilson Disease Protein. J. Phys. Chem. B 117: 13038-13050.
15- Mondol T, Åden J, Wittung-Stafshede P (2016) Copper binding triggers compaction in N-terminal tail of human copper pump ATP7B. Biochem Biophys Res Commun 470: 663-669.
16- DiDonato M, Narindrasorasak S, Forbes JR, Cox DW, Sarkar B (1997) Expression, purification, and metal binding properties of the N-terminal domain from the Wilson Disease putative Copper-transporting ATPase (ATP7B). J Biol Chem 272 (52): 33279-33282.
17- DiDonato M, Hsu HF, Narindrasorasak S, Que L, Sarkar B (2000) Copper-induced conformational changes in the N-terminal domain of the Wilson Disease copper-transporting ATPase. Biochemistry 39(7): 1890-1896.
18- Walker JM, Tsivkovskii R, Lutsenko S (2002) Metallochaperone Atox1 transfers copper to the NH2-terminal domain of the Wilson's Disease protein and regulates its catalytic activity. J Biol Chem 277 (31): 27953-27959.
19- Banci L, Bertini I, Cantini F, Massagni C, Migliardi M, Rosato A (2009) An NMR study of the interaction of the N-terminal cytoplasmic tail of the Wilson Disease protein with copper (I)-HAH1. J Biol Chem 284 (14): 9354-9360.
20- Larin D, Mekios C, Das K, Ross B, Yang AS, Gilliam TC (1999) Characterization of the interaction between the Wilson and Menkes disease proteins and the cytoplasmic copper chaperone, HAH1p. J Biol Chem 274 (40): 28497-28504.
21- Hasan NM, Gupta A, Polishchuk E, Yu CH, Polishchuk R, Dmitriev OY, Lutsenko S (2012) Molecular Events Initiating Exit of a Copper-transporting ATPase ATP7B from the *Trans*-Golgi Network. J Biol Chem 287 (43): 36041-36050.
22- LeShane ES, Shinde U, Walker JM, Barry AN, Blackburn NJ, Ralle M, Lutsenko S (2010) Interactions between copper-binding sites determine the redox status and conformation of the regulatory N-terminal domain of ATP7B. J Biol Chem 285 (9): 6327-6336.
23- Rodriguez-Granillo A, CrespoA, Wittung-Stafshede P (2010) Interdomain interactions modulate collective dynamics of the metal-binding domains in the Wilson Disease protein. J Phys Chem B 114: 1836-1848. doi: 10.1021/jp909450x
24- Ralle M, Lutsenko S, Blackburn NJ (2004) Copper transfer to the N-terminal domain of the Wilson disease protein (ATP7B): X-ray absorption spectroscopy of reconstituted and chaperone-loaded metal binding domains and their interaction with exogenous ligands. J Inorg Biochem 98: 765-774.
25- Rodriguez-Granillo A, Crespo A, Wittung-Stafshede Pernilla (2009) Conformational dynamics of metal-binding domains in Wilson Disease protein: molecular insights into selective copper transfer Biochemistry 48: 5849-5863. doi: 10.1021/bi900235g
26- Tsay MJ, Fatemia N, Narindrasorasaka S, Forbesc JR, Sarkar B (2004) Identification of the ''missing domain'' of the rat copper-transporting ATPase, atp7b: insight into the structural and metal binding characteristics of its N-terminal copper-binding domain. Biochim Biophys Acta 1688: 78-85 doi:10.1016/j.bbadis.2003.11.001
27- Wernimont AK, Yatsunyk LA, Rosenzweig AC (2004) Binding of copper (I) by the Wilson Disease protein and its copper chaperone. J Biol Chem 279 (13): 12269-12276. doi: 10.1074/jbc.M311213200
28- van Dongen EMWM, Klomp LWJ, Merkx M (2004) Copper-dependent protein–protein interactions studied by yeast two-hybrid analysis. Biochem Biophys Res Com 323: 789-795. doi:10.1016/j.bbrc.2004.08.160
29- Yatsunyk LA, Rosenzweig AC (2007) Cu (I) Binding and transfer by the N terminus of the Wilson Disease protein. J Biol Chem 282 (12): 8622-8631. doi: 10.1074/jbc.M609533200
30- DiDonato M, Zhang J, Que, L, Sarkar B (2002) Zinc Binding to the NH2-terminal domain of the Wilson Disease copper-transporting ATPase. J Biol Chem 277 (16): 13409-13414. doi: 10.1074/jbc.M111649200

31- Hou ZJ, Narindrasorasak S, Bhushan B, Sarkar B, Mitra B (2001) Functional analysis of chimeric proteins of the Wilson Cu(I)-ATPase (ATP7B) and ZntA, a Pb(II)/Zn(II)/Cd(II)-ATPase from Escherichia coli. J Biol Chem 276 (44): 40858-40863. doi: 10.1074/jbc.M107455200

32- Huang Y, Nokhrin S, Hassanzadeh-Ghassabeh G, Yu CH, Yang H, Barry AN, Tonelli M, Markley JL, Muyldermans S, Dmitriev OY, Lutsenko S (2014) Interactions between metal-binding domains modulate intracellular targeting of Cu (I)-ATPase ATP7B, as revealed by nanobody binding. J Biol Chem 289 (47): 32682-32693.

33- Lutsenko S, Petrukhin K, Cooper MJ, Gilliami CT, Kaplan JH (1997) N-terminal domains of human copper-transporting adenosine triphosphatases (the Wilson's and Menkes Disease Proteins) bind copper selectively *in vivo* and *in vitro* with stoichiometry of one copper per metal-binding repeat. J Biol Chem 272 (30): 18939-18944.

34- Sharma S, Rosato A (2009) Role of the N-Terminal tail of metal-transporting P1B-type ATPases from genome-wide analysis and molecular dynamics simulations. J Chem Inf Model 49: 76-83.
